# Employment and financial experiences in millennial family caregivers

**DOI:** 10.3389/fpubh.2026.1670668

**Published:** 2026-03-04

**Authors:** Megan C. Thomas Hebdon, Galilea Dupree, Janice Hernandez, Virginia Gallagher, Carolyn Phillips, Amy Patten, Neil Peterson, Michael Thomas

**Affiliations:** 1 College of Nursing, University of Utah, Salt Lake City, UT, United States; 2 Oncology Unit, St. David’s Healthcare South, Austin, TX, United States; 3 School of Nursing, The University of Texas at Austin, Austin, TX, United States; 4 Department of Neurology, University of Virginia, Charlottesville, VA, United States; 5 College of Nursing, Brigham Young University, Provo, UT, United States

**Keywords:** employment, family caregivers, financial well-being, Millennials, qualitative, social ecological model

## Abstract

**Introduction:**

Millennial caregivers comprise 25% of the family caregiving population, and they have unique financial and employment challenges due to life stage and generational experiences. The purpose of this study is to understand financial and employment experiences of Millennial family caregivers.

**Methods:**

This qualitative descriptive study uses secondary data obtained from two qualitative descriptive studies of Millennial family caregivers, one study focusing on the overall Millennial caregiver population and one focusing on the Latino population. For both studies, caregivers were recruited locally and nationally. Semi-structured interviews, open-ended responses from surveys, and focus groups were analyzed using thematic analysis.

**Results:**

Participants (*N* = 70) were predominantly women (71%, *n* = 50), White (59%, *n* = 41), and Latino (47%, *n* = 33), most caring for adults (79%, *n* = 55) and a portion caring for children (21%, *n* = 15). Eight main themes and one meta-theme emerged, aligning with the social ecological model. The meta-theme of identity was threaded through the other main themes including employment and career development; insurance and benefits; systemic barriers; daily living costs; healthcare costs; stress, strain, and struggle; interpersonal relationships and isolation; and support services.

**Discussion:**

The findings highlight the deep impact of employment and financial experiences on every dimension of Millennial family caregivers’ lives. Research, clinical practice, and policy efforts should address both upstream and downstream interventions that can address the diversity of challenges these caregivers experience related to the intersection of family caregiving, employment, and financial need.

## Introduction

Millennial family caregivers are those born between 1981 and 1996 (ages 29–43 in 2024) who are caring for a family member, friend, or neighbors with chronic health conditions (1). The term “family” often denotes a legal or kinship relationship, we deploy a broad definition of family that includes family of choice, which aligns with the American Geriatrics Society use of family caregiver (2). Efforts have been made in recent years to understand the needs and experiences of Millennial family caregivers (3, 4). Increased attention has been focused on Millennial family caregivers because relative to older generations of caregivers, this cohort has elevated rates of emotional distress, chronic health conditions, decreased financial resources, and greater time scarcity due to competing career and family demands, which often include raising young children (1, 5–9). Prior to that, most data came from population level studies focused specifically on Millennials or Millennial caregivers (1, 6–10). Collectively, these studies highlight that this generation of caregivers have needs related to employment support, financial resources, and social connection Therefore, the purpose of this study was to gain greater insight into the employment and financial experiences of Millennial family caregivers using secondary qualitative analyses of interviews with the general and Latino Millennial family caregiver populations. While Latinx is often used as a preferred term in academic settings to promote inclusive terminology, we have received feedback from our study participants who prefer Latino.

Caregivers in this generational cohort are in the stage of early and middle adulthood. This is a key developmental period where individuals are navigating training and schooling, career development, establishing intimate partnerships, raising children, purchasing homes, and saving for retirement (11). Family caregivers in this stage have the added layer of caregiving responsibilities, whether for aging parents or grandparents, partners, children, other family, friends, or neighbors (1, 8). Not only do these responsibilities demand more time and energy, but they can also add more financial demands (12–14). In some disease areas, such as cancer and dementia, this is referred to as financial toxicity, or financial problems related to the cost of medical care (15). For example, family caregivers invest over $7,000 per year to support individuals with chronic illness and disabilities (16). In addition, these family caregivers are often faced with the difficult decision of having to change jobs or leave the workforce, if their employers are not able to accommodate their family caregiving demands (17). This can lead to diminished current and future incomes, loss of employer-based insurance in the United States, decreased contributions to Social Security, and retirement savings stagnation or depletion (13, 14).

Generationally, these caregivers have faced significant challenges to their mental health with higher rates of loneliness and poorer overall health at the same age as Gen Xers (6, 7). While the reasons for this are unclear, some posit that this is related in part to decreased economic security (7, 18) In addition, this generation has weathered the 2008 and COVID-19 Economic Recessions, which have significantly impacted their achievement of adult milestones related to economics such as marriage, childbearing, home ownership, and retirement savings (11, 19, 20). Some issues that might contribute to these delayed milestones include higher student debt, wage stagnation, and inflation (21).

**Figure 1:**
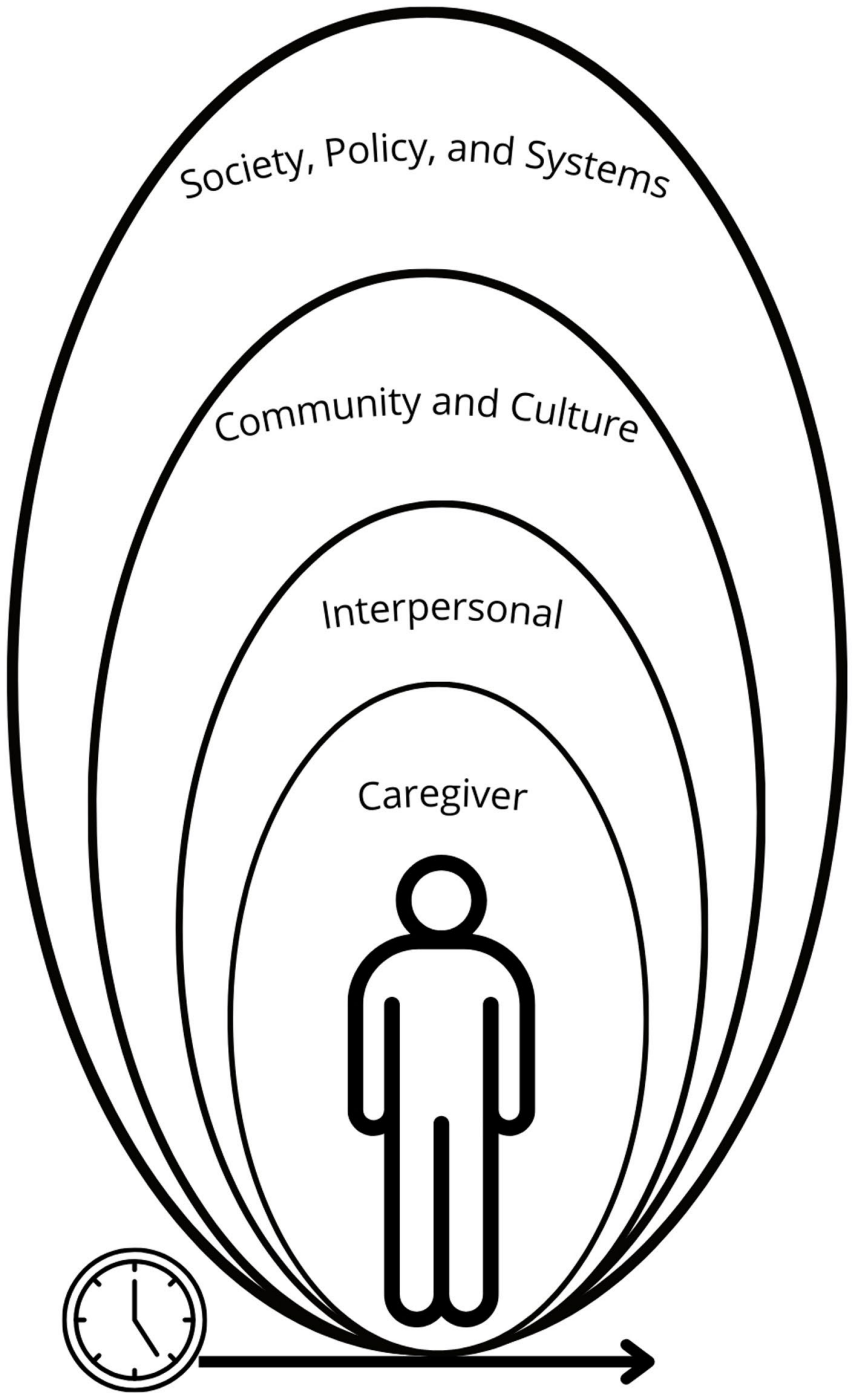
Social ecological model and family caregiving.

We used the Social Ecological Model as a framework for analyzing and interpreting our study findings, using a hybrid approach that is inclusive of inductive and deductive reasoning (22, 23). The Social Ecological Model frames the environmental structures that impact an individual’s health. We acknowledge that there may be some fluidity within the levels of the social ecological model due to the interactive nature of structures. Structures relevant to the experiences of family caregivers include interpersonal relationships, community, culture, society, policy, systems, and time. Interpersonal relationships for family caregivers would include care recipients, family, and friends that may be supportive of the caregiver’s needs. At the community level, there may be structural sources of support, including employers, and an expanded social network to meet family caregiver needs. There may also be barriers to accessing these such support. At the levels of culture, society, policy, and systems, the power imbalances related to wealth, healthcare access, and social capital start and trickle down to the individual experiences of the daily caregiver (22, 23). For example, women and minoritized individuals are more economically vulnerable and less likely to be employed in positions that have FMLA protections (24). This economic and job insecurity compounds existing health disparities that exist for these populations when combined with caregiving (17, 25). The concept of time means that caregivers’ employment and financial needs may not only be impacted in the present, but there may be ongoing impacts over time with retirement and career progression (see Figure 1).

The current study focused on financial and employment experiences of Millennial family caregivers. These issues have been acknowledged in prior research findings or in specific family caregiving contexts such as dementia and cancer, but a focused analysis on the experiences across caregiver contexts for Millennial family caregivers has not been addressed (4, 26, 27). This secondary analysis fills an important research gap on a widely acknowledged topic of concern, family caregiver employment and financial experiences, which significantly impacts long-term health trajectories for these family caregivers (28, 29).

## Materials and methods

This is a secondary qualitative descriptive analysis, answering a new research question with existing data, using data from two qualitative studies of Millennial family caregivers (30, 31). The first study, deemed exempt by the University of Utah Institution Review Board (IRB), focused on the stress and supportive care needs of the broad Millennial family caregiver population (4). The second study, approved by the University of Texas at Austin IRB, focused on the experiences and needs of Latino Millennial family caregivers (26). Continued use of the data for further analysis was deemed exempt by the University of Utah IRB.

Participants for both studies were recruited using online recruitment strategies. The first study used a research recruitment platform, ResearchMatch, as well as the senior author’s personal social media platforms to recruit Millennial family caregivers. Participants who met the following study criteria were provided with a study cover letter: born between 1981 and 1996, providing 10 or more hours of care and support to an individual with a chronic illness or disability. Agreement to proceed with the study was deemed as consent. For both studies, screening procedures were used to identify fraudulent responses, with 21 participants flagged and 11 participants excluded for meeting fraudulent response criteria (see Appendix 1). Participants recruited for the second study included individuals from a research database of previous research participants who agreed to be recontacted, community-based groups, national caregiving groups, and social media platforms (Facebook and Instagram). Study criteria mirrored the first with the additional criteria of identifying as Latino. Participants provided signed consent through REDCap following the provision of consent information. Further details about both studies can be found in the original published studies (4, 26).

### Data collection

For the first study, qualitative data from open-ended response surveys questions (42 survey responses) collected through REDCap and individual semi-structured interviews via Zoom (15 individual interviews) (see Appendix 2 for survey and interview questions). Interviews were recorded and transcribed verbatim. Interviews and surveys were de-identified and stored in a password protected file only accessible by members of the study team. The content of the questions for study 1 with both the survey and interview questions focused on an overall description of someone’s caregiving situation and responsibilities, the stressors and rewards of caregiving, responsiveness of health care team members, the impact of employment on caregiving and support from work, and strategies for managing stress both short- and long-term. For the second study, data were collected using 5 focus group interviews (*n* = 28) and one individual semi-structured interview via Zoom (total participants across both studies = 70; see Appendix 2 for focus group questions). The same process was followed to manage and store data to protect participant confidentiality. The focus group interviews and semi-structured interview for study 2 did not have specific questions about work or the responsiveness of health care team members and had more in-depth questions about support needs of caregivers (see Appendix 2 for more details). Demographic data for both studies were collected using REDCap surveys. For study 2, there was a limited response to the comprehensive demographic survey. Prior to focus group interviews, preliminary demographics were collected with a REDCap intake form including gender, age, race/ethnicity, and care recipient diagnosis. These were used due to incomplete comprehensive demographic surveys.

### Data analysis

A hybrid deductive and inductive approach to qualitative thematic analysis, as outlined by Braun and Clark (32) and guided by Sandelowski’s (31, 33) approach to qualitative descriptive methodology, was used to identify and analyze excerpts from both studies that addressed employment and/or financial experiences of Millennial family caregivers. This systematic process includes becoming familiar with the data, generating initial codes, searching for themes, reviewing themes, and defining and naming themes (32). An initial text search using a word list collaboratively developed with 3 team members (major categories: employment, family relationships, healthcare, money, insurance, aid) was used to identify excerpts, followed by line-by-line coding to support the coding process (34). Data from both studies were integrated and coded together, and each interview and survey were reviewed by two members of the research team. Contextual content supported the decision to include data in coding, with the requirement that it related to some dimension of the participant’s financial and employment experiences within the context of the Social Ecological Model.

Initial codes and themes were identified iteratively through review of data individually and with the team. To represent the depth and diversity of experiences among family caregivers across themes, sub-themes of challenges and needs were identified and the many issues that were found within these sub-themes were explicated, with the Social Ecological Model as a framework for finalizing themes. We sought to remain close to the data to represent participants’ perspectives through data analysis without overlaying extensive interpretation, which is in alignment with qualitative descriptive methodology (35). Due to the nature of a fixed dataset with secondary data analysis, all data were analyzed. Thematic saturation across both datasets was achieved, with no additional thematic elements identified (36). Trustworthiness was maintained using data triangulation (multiple data sources—interviews, surveys, and focus group interviews), researcher triangulation, an audit trail, and reflexivity during the coding process (37). Of those who completed data analysis, two coders identify as Latina, one is a Millennial, and all three have experience as family caregivers. From the larger team, individuals identify with the caregiving, Millennial, and/or queer communities. Both men and women are represented on the team. These identities were bracketed and addressed during the analysis process by each individual researcher and during analysis meetings to promote reflexivity. If there were disagreements about coding, the team reviewed their positionality, the data, and came to consensus (37).

## Results

Participants were primarily women (71%, *n* = 50), White (59%, *n* = 41), and Latino (47%, *n* = 33). Most were caring for adults (partner, sibling, parent, grandparent; 79%, *n* = 55) and 21% (*n* = 15) were caring for a child. Caregivers provided care for individuals experiencing a range of conditions including Type I and Type II diabetes, heart disease, cancer, Alzheimer’s and other dementias, stroke, epilepsy, autism and other developmental disorders, genetic disorders, anxiety, depression, and other health conditions and/or disabilities.

Eight main themes were identified that align with the levels of social ecological model including: employment and career development; insurance and benefits; systemic barriers; daily living costs; healthcare costs; stress, strain, and struggle; interpersonal relationships and isolation; and support services. Many of these issues crossed levels outlined by the social ecological model in terms of influence and impact. In addition to these themes, we noted a meta-theme of identity that was an overlay for these family caregivers with their employment and financial experiences that were described within the eight main themes. We have identified participant quotes by study (S) and interview (I)/focus group (G)/survey (S) (please see Table 1 for Themes and Exemplar Quotes).

**Table 1:** Exemplar quotes.

Theme	Quotes
Identity	I work really hard to provide us with insurance, and they automatically assume that I have Medicaid, which—there’s nothing wrong with that, but because of the color of our skin and where we live at they automatically assume I have Medicaid (S1 I4).
Employment and career development	Needs: Figuring out how to overcome crippling debt and dismal career outlook (S1 S67). Challenges: My last job thought they had complete disposal of my time, and had no concern for how their situation impacted my caregiving (S1 S7).
Insurance and benefits	Needs: Also, we recently switched to unlimited PTO. This has taken an IMMENSE load off of my shoulders (S1 S16). Challenges: My previous employer had counseling available to help me navigate our situation (S1 S24).
Systemic barriers	Needs: And then lacking the societal support, when things happen and go wrong it’s catastrophic when you cannot do those things anymore (S1 I9). Challenges: It is quite expensive when you do not qualify for any aid with that (S1 I14).
Health/healthcare costs	Needs: A professor in my college was offering free counseling so I decided to sign up for it. I was skeptical because I thought it was gonna cost extra money, but he told me it was for free that he noticed that a lot of students were going through a lot, especially after COVID (S2 G3). Challenges: But without insurance, basically we get to a certain point in the system and the doctor’s like, “Well, you need this procedure or you need these tests.” But you cannot afford them and you do not have insurance, so we are kind of in a holding pattern until something breaks (S1 I5).
Daily living costs	Needs: Trying to balance, taking care of yourself, providing for yourself, providing for someone else (S2 G3). Challenges: Those with financial consistency, there’s housing covered, there’s insurance covered, there’s food covered and all of the other Maslow’s Hierarchy is handled, basic needs are met, et cetera. And then you can actually take a deep breath and whatever crisis it is that you are facing does not feel so overwhelming and will not domino out of control (Study 1 Int 9).
Interpersonal relationships	Needs: I definitely take advantage of the friends that I have from my career field that I was in with Social Services and friends from college who are working in the mental health field (S1 I14). Challenges: It can be challenging with other social relationships like going out with friends, because [I’m] so busy with work and family responsibilities with [my] husband and three kids and then caregiving (S2 G5).
Support services	Needs: Some have been very responsive. We are working with the Institute of Aging in Cleveland and their case workers (Study 1 Survey 38). Challenges: I guess for me personally one thing that would really help me out would be some sort of in-home assistance, but that’s really expensive (Study 1 Interview 14).
Stress, strain, and struggle	Needs: Nothing really. I do not have the time or money for therapy (S1 S37). Challenges: And I’m off work at 4 p.m., but I do work from home on some days of the week, and I have a daughter who’s 11 and my son is seven. So sometimes it’s just really stressful at times because, and you know, on top of all this, I have a pet dog, Husky (S2 G2).

First S: Study; I: Interview; Second S: Survey; G: Focus Group.

### Meta-theme: identity

Millennial caregivers discussed the interaction of systems and relationships with their identities in diverse ways, including age (being a young caregiver) and generational identity when navigating support services: “I think, again, for our age group, ours seem different than older adults. I think sometimes there are more protections for people in other age groups (S1 I1).” Professional identity was also identified as factor navigating interpersonal relationships, education, and employment: “And so when I met him a few years ago—I too am a nurse, so sometimes I have a hard time separating being a wife and a nurse” (S1 I2). Caregivers who were women discussed the societal pressure they felt to fulfill the caregiving role which impacted their interpersonal relationships and stress levels:

As a woman, as a wife, as a mother I think that there’s a high societal pressure that is placed on us to take on the emotional wellbeing of other individuals in our lives and we are expected to shoulder that (S1 I9).

One caregiver described the decision to disclose disability status with employment:

We have to continuously fill out these voluntary disability things and every single time, I’m like “What do we say?” Like, I’ve always avoided saying yes because even though—sometimes what I have is listed because I’m like “Well, I don’t know if there is going to be…” Like, they say there’s not going to be discrimination, but—just having someone to explain the discrimination policy (S1 I1).

Socioeconomic status was identified as a systemic barrier for caregivers to meet their daily living costs: “When you’re that far below the poverty line, yeah, it’s just you get rent and then you see where you are with everything else” (S1 I5). Participants described racial, ethnic, and immigrant identities that impacted their caregiving, navigation of support services, and interpersonal relationships:

“It isn’t easy for most of us who are Latinos to move on with life… she [mother] had to come over here to start a fresh new life for herself. So, it wasn’t really easy to fit in. It took me about fifteen years to, so I try to do that for my daughter (S2 I6).

Interpersonal relationship dynamics related to family, cultural, and personality also impacted their motivations and experiences with caregiving. One caregiver described their independence due to not having external support: “I really value independence and a lot of that comes from the fact that I have not been able to count on any external aid” (S1 I5). Another caregiver described their commitment to caregiving due to intergenerational responsibility:

I’m giving back to my parents and making their lives a bit easier for a change…they have given me and my sister their livelihoods and it’s my opportunity to do the same for them (S1 S67).

### Employment and career development

Caregivers described multiple *needs and challenges* related to employment and career development. In terms of *needs*, they described the importance of supervisor, colleague, and organizational support:

They are sympathetic and supportive, and have oftentimes covered for me, rescheduled meetings and adjusted my responsibilities and/or deadlines. One supervisor has been very accommodating of the last-minute changes that come up with caregiving. There are instances though when they maintain the same expectations and workload because of the nature of the position (S1 S79).

While still facing difficulties balancing caregiving with work, caregivers described the benefits of having a predictable income and job stability, and for remote or flexible work:

Well, I’ve had to split up my day now. So instead of just working on the regular hours like eight to even or maybe seven to six, or whatever it is during regular hours, now it’s split up. So, I go in in the morning, then I go take care of my grandma. And then I work a little home. Then I go back and take care of her some more, and then I go back home to work (S1 I3).

Some caregivers described a cultural environment supportive of family caregiving, although still feeling vulnerable when asking for accommodations: “They’ve been very supportive. It is hard to be open about the issues, but they’ve really cultivated a positive and healthy work environment” (S1 S55). An example of vulnerability is this caregiver’s statement, “Boss says she understands but I’m just waiting for it to be used against me” (S1 S39). Another caregiver identified a perception from employers that devotion to family is perceived as a lack of professionalism:

They understand and are accommodating but definitely understand it to be indicative of my work ethic and with regard to my level of professionalism, rather than as what it is, which is a devotion to my family and their success…And that’s where my loyalty lies- with my family who I love and will be with the rest of my life. Not with the corporation that barely provides insurance, and I feuded to promote from within (S1 S62).

Caregivers described *challenges* such as student debt, stagnant wages, having to work multiple jobs, and difficulty balancing caregiving with career growth and education:

I did have to drop out of grad school as I cannot complete student teaching during the pandemic with a medically complex toddler plus the stress was just too much (S1 S20).

Finally, workplace issues such as work productivity/absenteeism/presenteeism, losing or changing jobs, reducing hours, fearing unemployment, and age expectations related to caregiving and work were also noted. This caregiver described job instability due to schedule disruptions from family caregiving, “I am generally told they cannot accommodate my schedule needs or when my daughter gets ill & is hospitalized, I lose the job” (S1 S48).

### Insurance and benefits

With insurance and employer-provided benefits, caregivers described *needs* such as state paid family leave, having support at work that is more than lip-service, and having comprehensive and accessible paid time off, the Family and Medical Leave Act (FMLA), unemployment and disability benefits. This caregiver described the security provided by having FMLA: “trying to balance work & caring for my son & mother, I don’t have any PTO & if it wasn’t for FMLA I wouldn’t have my job” (S1 S39). Another caregiver reflected this same need, but more in-depth challenges accessing their employer-based benefits:

Intermittent PFL[paid family leave]/FMLA and information from human resources, working from home—but that is only because of the pandemic, I was denied the same accommodation request for remote working roughly a week before everything shut down (S1 S79).

Some caregivers noted *challenges* such as having no insurance due to employment status: “I have to cover what I felt insurance would cover for me, I had to cover it all by myself, because I do not have a job to pay insurance fees” (S2 G3). Others noted issues related to having no leave benefits: “I have no paid time off, if I miss anytime, I do not get paid which is difficult as I am the sole financial provider for my household” (S1 S39).

Additional health insurance issues were noted including being under-insured, having limitations in care they can access without insurance, not being able to put an aging care recipient on their employer-based insurance, and paying for expensive care out-of-pocket that is not covered by insurance. This caregiver noted:

We have not had the best luck with medical providers or insurance coverage, so I’ve spent the past year learning how to be an advocate. I have filled out 4 hospital financial aid applications, filed an appeal to health insurance for not covering a $11 k bill for emergency seizures, applied for Medicaid 5+ times, etc. (S1 S20).

### Systemic barriers

Multiple systemic barriers were identified as caregivers described their needs and challenges. One caregiver summarized their overall *needs that addressed health policy, state, and community programs*:

A universal basic income and universal basic healthcare. On a community level or a state level, wraparound programs that could benefit individuals and communities and stuff, making sure that preschool programs and education is covered, expanding on school, public schools and higher education, just making sure that food is not a scarcity, housing (S1 I9).

The quote above highlights *needs* such as easier access to services across the board, having more structural community/society support for both the caregiver and care recipient, and having less housing and food scarcity. One caregiver identified the presence of a transportation service, but limitations in access:

I feel like the hospital offers free rides, but they don’t do it, and then there’s a Handi-Van, but they don’t do it, so it’s just like the more you know, the better off you are, so we found out that the Quality Cab will push him up if he takes their wheelchair van, so I just feel like there’s little things that are so hard for us to figure out, but there’s easy answers out there I guess (S1 I14).

This caregiver described the difference in systems and support based on geographical location: “But we are in Arkansas and it’s not exactly—we are a little behind here. We’re a third-world country or third-world state inside of the United States. (S1 I13).

Many caregivers reported *challenges* related to difficulty navigating the system. One caregiver directly stated: “I have never benefited from any government grants or donations” (S2 G3). Other caregivers noted hours spent trying to get the care they need:

Because we moved, he needed to get a new neurologist and we were on an HMO plan and our primary care physician like gave us a 60-day like authorized referral and the office that she worked at just like wasn’t calling and wasn’t giving the referral to the office that we need to go to. So, I was like spending literally like probably ten hours a week on the phone with them and making no progress for the past month and finally, we have an appointment for the Epilepsy Center for him (S1 I1).

Paperwork was a prominent barrier for individuals: “It took seven and a half months to get my wheelchair, like my personalized wheelchair because of a piece of paperwork and I was just like ‘This is ridiculous’” (S1 I13). These challenges related not only to the needs of care recipients, but also caregivers. For example, one caregiver could not access needed mental health support due to insurance issues:

I haven’t gone anywhere in a couple years. I started going after my dad had his first leg amputated, and then I stopped going because of insurance issues, but I really, really, really want to go back. I’m just waiting for my insurance to switch again for the better so that I don’t have to pay so much to go, but that’s a top priority of mine once that happens, because I feel like I need it. It’s a lot to live this life, and you know that (S1 I14).

### Healthcare costs

Caregivers described the *need for more support* for their own healthcare, and the *challenge of health care costs*. When describing their own healthcare, caregivers noted the tension between their own needs and that of the care recipient: “I have to put my own injuries aside to take care of hers” (S1 S51). One caregiver described deferring care, due to time and financial demands, “I know I need to have therapy, but we can’t afford it right now nor do I feel like I have enough time for it” (S1 S20). Another caregiver described the need for childcare, so they could access care for themselves: “Right, or even healthcare for myself. <laughs> If there was a place I could take my kids on-site—because we’re not gonna sign-up for daycare just for that” (S1 I8). In summing up this issue, a caregiver stated, “I think for every caregiver they never get the cure they give to people for themselves” (S2 G3). In describing health care costs, caregivers noted challenges related to medication, medical supplies, dietary needs, and appointment scheduling:

“Budgeting for medical supplies, my husband keeping his numbers under control, making and going to necessary appointments, trying to eat right to keep his numbers in check, worrying about the future if he doesn’t start taking better care of his diabetes” (S1 S22).

Another caregiver noted the relief of having less costly medication, “So, one of the good benefits of it is the medicine itself is not too expensive. But I know that that’s one of the few blessings we’ve got here” (S1 I5).

### Daily living costs

Daily living costs were a significant concern for many family caregivers, especially considering caregiving demands. One caregiver described the *need for greater financial stability*, due to the stress of living day to day:

There are days where I wake up thinking—because I’ve been in situations over the last couple years where I have gone into work one day and they say “Oh, we don’t need you today,” and that day is another day of don’t have money coming in. So, I have to be in a situation where okay, how can I make sure I have money coming in that day? So, I have to resort to taking an odd and end job. Sometimes it’s something I don’t want to do, but I know if I don’t, I’m not going to be able to provide (S1 I7).

The tension between being available for caregiving responsibilities and maintaining a job to provide for daily needs and quality of life was a consistent challenge caregivers noted: “I know that I cannot quit my job, because if I do, they’ll be no way to pay the bills” (S2 G3). Another caregiver described, “I also taking upon myself as a responsibility to make any money I can on the side to just make our quality of life easier” (S1 I5). *Some challenges* noted included having no wiggle room financially while working, providing daily living cost support to care recipients, managing the health of care recipients, and having inadequate time and not enough support. One participant stated that it is “the little things.” This caregiver described that it is “Stressful to worry about budgeting and if he’s keeping up with medical management as he should” (S1 S22). Another caregiver outlined additional costs related to caregiving, “Buying groceries, I mean cleaning her house. It’s like everything. Like I’ve basically taken over her life” (S1 I3).

### Interpersonal relationships and isolation

Interpersonal relationships had a significant impact on these family caregivers when navigating employment, caregiving responsibilities, and financial need. *When identifying their needs, met or unmet*, these caregivers described the importance of help from friends and family: “Coordinating with my family to avoid burn out” (S1 S46); and “My dad is a very calm person so usually just talking to him about what we are going through together” (S1 S80). Conversely, these relationships also created more *challenges*:

I could have worked a little bit longer, but with the amount of support they were needing, I wasn’t really able to give them the amount of support they needed and focus on school. I’m getting ready to start my clinical practicums, and so I just—I needed to be able to have some more devoted time to just support them, so I wasn’t really able to put everything I needed to into being a better speech pathologist, but I also wasn’t really focusing on my marriage either, and my husband’s, again, a very good sport, but I wasn’t able to provide other supports and put effort in where it was needed (S1 I6).

Another caregiver noted, “I have to do the caring for everyone, and most times I tend to overlook myself. I can’t remember when I barely bought anything for myself” (S2 I6). Lastly, a caregiver described *the challenge* of being caught between current circumstances and the chances of adding a child to a family that is already stretched thin:

I don’t want to be in a situation where God forbid we bring a child into this world. It’s not the life it deserves. Sure, adoption is always an option, but it’s like do I want to put that thought in there because I know I have a bleeding heart and I would want to keep it. It is not fair to put a child into a situation of being desolate (S1 I7).

In addition to interpersonal relationships, caregivers described feeling isolated and misunderstood with their family caregiving role and responsibilities: “No one really understands the need for me to be with my son rather than at work” (S1 S74). *Community as a vital need* for psychosocial and tangible support was discussed by many caregivers:

We come together and support each other. At times when we know that a member of ours is going through hard financial times, we give the little we have. Tell other people like, Let them assist us in every little way (S2 I6).

Caregivers described themselves alone as a finite resource, and the *challenge* of being the only one providing care: “There’s also only so much aid I can provide her using myself as a resource” (S1 I5). Caregivers noted the feeling of being alone in their challenges, learning through necessity to rely on themselves, and an invisibility with many illnesses that extends to financial challenges. One caregiver stated: “The most difficult thing about caregiving is isolation because most times it feels like I’m cut off from the outside world” (S2 G3).

### Support services

When discussing available support services within their community and health care organizations, caregivers noted the *need* for more care and support service coordination, accessible and affordable childcare, and greater support with financial challenges. One caregiver described the difficulty of navigating the system alone: “It is every man for themselves. I have set up everything with no help (PT, OT, speech therapy, physiatrist, neurologist, psychiatrist, counselor, etc.)” (S1 S44). Two caregivers remarked on the absence of support related to social needs within the work and health care sectors:

I wish that there was support networks in institutions like separate from the department you are applying to work with or whatever, where they just like honestly lay it out for you, like “These are your protections. This is what you do if something goes wrong” (S1 I1).

In referring to clinicians and the health system, this caregiver noted that they were “Somewhat responsive, mostly in regard to the medical needs (not social or support needs)” (S1 S53). In describing needed assistance, this caregiver identified the need for more financial support: “Financial since she doesn’t get any assistance” (S1 S51).

The *challenges* noted with support services included concerns about the degree of help that is offered based on caregiver sociodemographic characteristics:

I wear like my college sweater that is like a fancy school that I went to, which I usually never talk about, because I think that stuff does not matter at all, but I know like unconsciously it can have an impact on how a doctor perceives your intelligence (S1 I15).

Caregivers also described the challenge of both affording supportive care out-of-pocket and that care being trustworthy: “but childcare, especially childcare for a special-needs kid, is very expensive and hard to find trustworthy people” (S1 I8); and “I can’t afford to get like a caregiver to pay. So, I have to do it myself to save money and to be able to get her medications too” (S2 I6).

### Stress, strain, and struggle

Millennial caregivers noted the ongoing stress, strain, and struggle related to employment and financial issues. *When noting needs,* these caregivers described the need for self-care, but also the barrier of not having money for self-care: “Self-care is also helpful for me. I find that I need down time and quiet in order to process my feelings and experiences in order to be able to move forward” (S1 S20); and “Coping takes money and time, and I don’t have either” (S1 S21). Financial *challenges* on top of other responsibilities and caregiving demands were a common source of stress:

My major sources of stress are:—Financial stability (or lack thereof)—Lack of sanitary/cleanliness in my household as a result of my father’s bad habits (I have an autoimmune disease, and am high risk from and immune system perspective)—Not having enough hours in the day to care for myself, my two dogs, and my father, at times (S1 S16).

Finally, caregivers described the *challenge* of balancing work responsibilities while managing the unpredictable but inevitable health issues with the care recipient:

It will happen again, that sort of knowledge that this is not over, and I am going to have to deal with this again and who knows what the situation will be. I think that is most stressful in terms of anxiety inducing. I would say trying to balance like this new job, which already is sort of in this weird situation because of the pandemic with having to be home even more than like most people during the pandemic (S1 I1).

## Discussion

Our findings outline the complexity of experiences related to finances and employment for Millennial family caregivers. With the inclusion of data from both a broad and Latino Millennial caregiver population, we have a strong representation of a significant group of Millennial caregivers representing 27% of this caregiving population (1). All themes were found within both groups, but it is of note that there are additional systemic barriers for Latino individuals in the United States including racism, immigration, and health care access (38). In addition, study participants represented caregivers of individuals across the disease and age spectrum who are experiencing financial and employment difficulties. The contribution of this study is the focus on a discrete generational cohort, Millennial family caregivers. Due to both developmental milestones, generational norms, and historical events, these caregivers represent a unique group with distinct financial challenges (1, 11). Although Millennials constitute the largest generation in the workforce and a quarter of family caregivers in the United States, they are financially vulnerable (1, 9, 21, 39). This bears out with our qualitative findings with caregivers describing challenges and needs related to financial need and employment that are influenced by every layer of the Social Ecological Model.

The prominence of identity as a meta-theme highlights the intersectionality of caregiving across the levels of the Social Ecological Model (23, 40). Caregivers’ roles are influenced by generational, cultural, socioeconomic, and gendered dimensions, which shape their experiences and coping mechanisms, and may result in additive stressors when such experiences are shaped by power and access to resources. These findings align with prior research, which emphasizes the critical role of cultural identity and societal norms in shaping caregiving responsibilities and outcomes (41). Millennial caregivers, in particular, grapple with the tension between societal expectations of early and middle adulthood and their caregiving responsibilities, often feeling overwhelmed and under supported (8, 42). This generational cohort is also more diverse than previous generations in terms of racial, gender, and sexual identities (1). Due to historical policies impacting access to social and economic resources and ongoing gendered and racialized power differentials, these caregivers are at higher risk for poorer work and financial outcomes (40, 43). Addressing these power and identity-related challenges requires culturally tailored interventions and broader public recognition and policy-based decisions that support Millennial caregiver contributions. For example, Kim et al. (44) describe psychosocial phenotyping, as a way to provide tailored interventions that are reflective of individual internal and external contexts and needs.

Caregivers’ narratives around employment and financial challenges echo existing literature that highlights the adverse economic impact of caregiving (16). Many caregivers reported difficulty balancing work and caregiving responsibilities, compounded by stagnant wages, limited job flexibility, and insufficient employer-provided benefits (45, 46). These findings point to the urgent need for policies such as expanded paid family leave, affordable childcare, and workplace accommodations to support caregivers (47, 48). Organizations could also benefit from fostering a workplace culture that values and supports family caregiving responsibilities, as this has been linked to improved employee retention and productivity (17).

As discussed, financial toxicity is the perceived financial distress associated with health-related costs (15). Caregivers in our sample clearly highlighted perceived financial toxicity but went beyond the costs associated with health care to include every facet of their life that was affected by the intersections of caregiving, employment, and financial status. These sentiments included feeling vulnerable when asking for accommodations at work, feeling stressed by living expenses and care costs for both themselves and the care-recipient, and feeling strained by the lack of achievement and autonomy regarding employment. This exemplifies the importance of addressing financial and employment challenges with a wider lens, that encompasses all levels of the Social Ecological Model, including time. “Financial well-being” may provide a more accurate representation of these caregivers experiences, including present and future orientation and encompassing both security and choice (49). For example, a family caregiver with financial well-being would have control over daily financial obligations, be able to absorb unexpected financial hits, and have the financial flexibility to enjoy life and meet financial goals (49). Our findings suggest that there are many barriers to achieving financial well-being for Millennial caregivers, and there are significant relational and health impacts when financial well-being is not in place. While there are behaviors, beliefs, and skills that impact financial well-being, caregivers are also in social and economic environments that impact their financial and employment choices (21, 43). This move to financial well-being means that supporting Millennial caregivers requires a broader ecosystem approach that acknowledges the systems and contexts that influence financial well-being (12). Much of current research addresses the immediate financial impacts of family caregiving such as costs of healthcare, and social needs, but there are broader and deeper impacts that affect caregiver quality of life over time (50–52).

The financial and employment stressors described by caregivers in this study should be viewed in the context of significant and real objective financial stressors that are specific to the multiple identities of caregivers in the Millennial cohort including (a) being a young- to middle-aged adult who may be career-building and family-building (53), (b) being a caregiver, which brings time scarcity associated with competing demands across care, family, and career (8), and (c) being a consumer during the current economic climate. For example, adults in their 30s in the U.S. are often under more financial strain than other age groups due to the costs of building careers, buying homes, and raising children (11, 54). Further, caregivers often shoulder the burden of increased financial costs, relative to non-caregivers, to support the person with the chronic condition with living expenses and/or care costs (12, 55). These objective costs are exacerbated by the current economic climate in which housing, daycare, adult care, and transportation costs relative to average income are all at historical highs (20, 56, 57). These realities may be useful in a therapeutic context to remind Millennial caregivers that there are historical and contextual forces affecting their financial and employment experiences.

### Research priorities

Our findings suggest the importance of a social ecological perspective that supports Millennial caregivers with their employment and financial needs (22, 23). Research should address both upstream (policy-based and larger community) and downstream (individual and family) interventions to support caregivers in their employment, resource access, systems navigation, family responsibilities, and financial planning. Most interventions are focused on the care recipient or care recipient-caregiver dyad and may leave out the other family responsibilities that impact caregiving, employment, and financial stability (51, 58). An immediate priority is taking a comprehensive assessment of caregiver employment and financial needs that transcends treatment-related costs, because these issues are often not addressed in current interventions.

### Clinical practice recommendations

Clinically, a minimum intervention would be conducting social needs assessments, but caregivers may still struggle financially even if all their social needs are met (59). Health care decisions should be made in the context of caregivers’ existing financial resources, and caregivers should be connected to programs and resources that can augment these resources. For example, access to affordable high-quality care for children and older adults would support Millennial caregivers with their caregiving, parenting, and work responsibilities.

### Policy recommendations

Finally, the needs of caregivers are well known, but there remain significant policy gaps in the United States related to caregiver financial needs. A starting point would be universal paid family leave that would allow caregivers to have an income while meeting caregiving obligations (60–62). Some states, Medicaid, and the VA system have caregiver reimbursement programs that could be broadened to support the care provided by caregivers that results in cost savings to the health care system (14). Globally, there are examples of caregiver support programs and policies including paid family leave, ongoing respite programs, tax credits, and caregiver education programs (63, 64).

### Limitations

Our study has limitations including online recruitment bias, limitations of secondary data analysis, the use of two qualitative datasets with a general Millennial population and a Latino Millennial population. Online recruitment may result in bias and fraud. We implemented a fraud protocol to address bots and other sources of fraudulent results. We may have done so imperfectly and acknowledge that as a limitation. There may be other online recruitment issues that favor individuals with access to the internet and algorithmic issues that are outside of the researcher’s control but may still result in a biased sample. While both studies focused on the needs and experiences of these populations, the questions in the interviews for both studies are not a perfect mirror (See Appendix for questionnaires). Question prompts yield unique findings based on the prompt, therefore there may be differences in the data. This could lead to overrepresentation of findings within one dataset over the other. Interestingly, the themes were consistently found within both datasets, although there were more discussions on the impact of immigration in the Latino population. Incidences of bias and racism were described by participants in both studies. Despite this, our study findings should be interpreted with the limitations outlined above.

In addition to the above limitations, there are issues related to demographic representativeness, geographic transferability, and the time-bound nature of our findings. Our participant population was primarily White and women, which does not reflect the broader Millennial family caregiving population in the United States which is almost evenly split between men and women and is more demographically diverse (1). We also have data from a broad Millennial caregiver sample and a Latino Millennial caregiver sample. About 27% of Millennial caregivers are Latino, making them the largest minoritized group within this caregiver generational cohort (1). Studies often have an overrepresentation of non-Latino participants, where we have an overrepresentation of Latino participants. This may lead to transferability issues but could also be a strength with having greater representation of a group that faces systemic barriers to job, resource and healthcare access (38). This study was conducted in the United States with its unique context and policies related to employment and financial well-being. While our study was conducted with individuals across the United States, specific regional differences were not examined. These geographical issues also limit the transferability of our findings. Future research should use purposive sampling to obtain a more balanced representation of gender and race and should consider regional and cross-national perspectives about employment, financial well-being, and caregiving.

Our interviews were conducted cross-sectionally, which does not allow for experiences that may change over time. We also used a disease agnostic approach to recruitment, but did not examine differences in care recipient conditions and the impact on financial well-being and employment. These are all important considerations for future research efforts.

## Conclusion

Millennial caregivers are a large and critical group that are contributing to the social and economic well-being of the United States through their caregiving, family, and work roles. They are at risk for poor economic and health outcomes related to their family caregiving roles which may compound over time. Our study findings suggests that their financial well-being and employment challenges and needs operate at all levels of the Social Ecological Model, meaning that this is not only an individual issue, but a societal issue. Our study strengths include a sample with care recipients at different ages and with different chronic illnesses, a large number of Latino Millennial caregivers, and the use of the Social Ecological Model to frame the analysis and findings. This model is salient in addressing the employment and financial challenges of Millennial family caregivers through research focused on the broad impact of caregiving on employment and financial well-being, clinical practice changes that acknowledge the life stage and needs of Millennial family caregivers, and policy interventions, such as paid family leave, that acknowledge the dynamic nature of balancing caregiving with work responsibilities.

## Data Availability

The data analyzed in this study is subject to the following licenses/restrictions: data are available upon request. Requests to access these datasets should be directed to Megan Thomas Hebdon, meg.hebdon@utah.edu.
