# Supplementary material for: Employment and financial experiences in millennial family caregivers
Source: Front Public Health. 2026 Mar 4;14:1670668. doi: 10.3389/fpubh.2026.1670668 (PMC12995795; doi:10.3389/fpubh.2026.1670668)
Supplement: Supplementary file 1 [file Supplementary_file_1.docx]

Appendix 1: Fraudulent Response Protocol

1.     Add a Captcha to the survey.

2.     For screening questions, require text entry for all questions.

3.     Review survey entries for the following characteristics:

a.     Is over half of the survey incomplete? (1 point)

b.     Is the email address provided unusual (e.g. long string of numbers or nonsense letters)? (1 point)

c.     Did it take the participant less than 5 minutes to complete the survey? (1 point)

d.     Does the email match or closely match a prior submission? (1 point)

e.     Is there conflicting information in survey responses? (1 point)

f.      Are responses to questions incongruent or unclear? (1 point)

4.     If score of 1, the participant receives an email listed under potentially fraudulent. If score of 2 or greater, the participant receives an email listed under fraudulent.
