# Supplementary material for: Employment and financial experiences in millennial family caregivers
Source: Front Public Health. 2026 Mar 4;14:1670668. doi: 10.3389/fpubh.2026.1670668 (PMC12995795; doi:10.3389/fpubh.2026.1670668)
Supplement: Supplementary file 2 [file Supplementary_file_2.docx]

Appendix 2: Survey, Interview and Focus Group Questions

Study 1 Survey Questions

Please describe your caregiving situation:

How does caregiving impact your other responsibilities?

Please outline an average day including caregiving and your other responsibilities.

What is most stressful about your caregiving role?

What is most rewarding about your caregiving role?

What are the major sources of stress for you right now?

How responsive have health care providers (doctors, nurses, social workers, therapists) been to your needs as a family caregiver?

How has your employment been affected by your caregiving responsibilities?

What kind of support is available or has been available at work to help you manage caregiving and your job?

How have your boss and co-workers responded to your caregiving responsibilities?

What do you do to handle stressful moments related to caregiving or your other responsibilities?

What do you do to handle the long-term stress of caregiving and your other responsibilities?

What has been most helpful for managing stress?

Study 1 Interview Questions

To start, please tell me about your caregiving situation, including the good parts and the hard parts:

What are your other responsibilities, including work, family, social life, religious involvement?

Between your other responsibilities and caregiving, what tends to be the most stressful?

How responsive have health care providers (doctors, nurses, social workers, therapists) been to your needs as a family caregiver?

Have you ever felt judged by health care providers (doctors, nurses, social workers, therapists)? If so, how?

How has your employment been affected by your caregiving responsibilities?

What do you do to handle stressful moments related to caregiving or your other responsibilities?

What has been most helpful for managing stress?

What do you need to help you manage stress?

Thank you for all of the information you have provided. Your work as a caregiver is of utmost importance. We recognize all that you are doing, and we are grateful for your feedback today.

Study 2 Focus Group Questions

Thank you all for your willingness to participate in this focus group. We value your time and the work you do as caregivers, and we are very appreciative of your contribution to this study. Before we proceed, we want to review a few things. First, that your participation in this study is voluntary. This means that you do not need to answer any questions you are uncomfortable answering, and you can leave the focus group at any time. We will be recording the audio from this session, and a few researchers will be taking notes during the session. If you do not wish to be recorded, you are welcome to excuse yourself from the group. Do any of you have questions before we proceed?

Caregiver Experiences:

To get us started, can each of you tell us about your caregiving situation? We’ll start with. . .

We also want to know about the other things in your life you are doing besides caregiving. We’ll start with. . .

Thank you so much for what you have shared. Now, can you share what is most challenging about caregiving for you? We’ll start with. . .

Thank you. We also like to know about the good things that come from caregiving. Can you share any positive things that have come from your caregiving? We’ll start with. . .

Support Needs:

Part of this research is to understand what types of support caregivers like you need. Can you share what support you have received that has been helpful? We’ll start with. .

Has there been any support you have received that was not helpful? If so, please describe. We’ll start with. . .

Thank you. Now we want to know if there is support you feel like you need that you have not received? We’ll start with. . .

This is great information. We appreciate your willingness to share.

Coping:

Caregivers often have things they do to help them cope with caregiving and their other responsibilities. Can each of you share what you do to cope? We’ll start with. . .
